# The preservation and augmentation of volatile terpenes in cannabis inflorescence

**DOI:** 10.1186/s42238-020-00035-z

**Published:** 2020-09-14

**Authors:** Justin Bueno, Emily Leuer, Michael Kearney, Edward H. Green, Eric A. Greenbaum

**Affiliations:** Vireo Health, 1330 Lagoon Ave, 4th Floor, Minneapolis, MN 55408 USA

**Keywords:** Terpenes, Medical cannabis, Recreational cannabis, Novel packaging, Whole plant products

## Abstract

**Background:**

Terpenes contribute to the pharmacology, efficacy, aroma, and flavor of cannabis inflorescence, improving the experience for medical and recreational users. Terpenes are inherently volatile, resulting in the loss of terpene content as inflorescence ages. A method to establish and/or maintain a desired terpene content of cannabis inflorescence is needed. A novel packaging method was investigated for the preservation of native terpenes and the replenishment of terpenes to depleted inflorescence over various storage durations.

**Methods:**

Inflorescence samples from two different chemotypes (DJ’s Gold, Cream Caramel) were obtained from a state licensed medical cannabis organization. Samples from the DJ’s Gold chemotype were depleted of terpenes whereas samples from the Cream Caramel chemotype had a terpene content representative of inflorescence available for medicinal or recreational purposes. Inflorescence samples were stored using the novel packaging approach, in airtight containers in the presence of external terpenes. Control samples were similarly stored without external terpenes. Terpene content of the inflorescence samples were quantitively determined by headspace gas chromatography mass spectrometry (HS GC-MS) after various storage durations. Main effects analysis was used to determine the impact of various parameters on the effectiveness of the system.

**Results:**

All samples stored using the novel packaging approach had a higher terpene content than their corresponding control. 1.18% (w/w) of external terpene, relative to inflorescence weight, was the minimum amount required to maintain the initial terpene content of the inflorescence after 6 weeks of storage. Main effects analysis showed that augmentation of inflorescence terpene content was dependent upon the amount and type of external volatile utilized. The terpene profile of inflorescence samples from two separate harvests were selectively adjusted, reducing the percent difference of the two sample’s terpene profiles by 39.5%.

**Conclusions:**

A successful proof of concept was achieved for preservation, augmentation, and replenishment of terpenes to cannabis inflorescence over various storage durations. Inflorescence stored using the novel packaging approach is a significant step towards providing patients with cannabis inflorescence of reproducible and reliable terpene content, an important component of inflorescence efficacy. The novel approach for replenishment of terpenes to depleted inflorescence represents an exciting development for patients and manufacturers.

## Background

Terpenes are volatile phytochemicals which contribute to the characteristic aroma and flavor of cannabis. Moreover, terpenes possess relevant pharmacological properties including analgesic (Guimarães et al. 2013), anti-inflammatory (Cho et al. 2017), neuroprotective (Cho et al. 2017; Manayi et al. 2016), and anxiolytic characteristics (Linck et al. 2010). Terpenes also have the potential to enhance the pharmacology of cannabinoids and other phytochemicals characteristic of cannabis (Russo 2011; Ferber et al. 2020; Zhang et al. 2017). The so-call “entourage effect”, or the theory that whole plant products are more effective than their individual chemical constituents, can influence the efficacy, pharmacological, and euphoric effects of cannabis use. These are important factors for both medical and recreational cannabis users. Over 200 terpenes have been identified in various chemotypes (strains) of cannabis (Roy and ElSohly 2014). Although there are combinations of terpenes considered characteristic of specific chemotypes (Elzinga et al. 2015), the terpene profile is dependent upon many factors including; genetics, inflorescence age, and environmental, cultivation, and harvest conditions (Fischedick et al. 2010; Potter 2009). Furthermore, a lack of standardization means that a given chemotype cultivated in one location, may yield inflorescence of varying chemical composition as compared to the same chemotype cultivated in another location or at a different time. Loss of terpene content post-harvest is well established. Ross and ElSohly measured a 31.0, 44.8, and 55.2% loss of terpene content in *Cannabis sativa* inflorescence which had been air dried and stored for 1 week, 1 month, and 3 months, respectively, as compared to freshly harvested inflorescence (Ross and ElSohly 1996). All these factors lead to both inter- and intra-harvest variability in terpene content and present challenges for patients and consumers seeking consistent efficacy and experience from use of cannabis inflorescence.

We recently reported a method for the preservation of volatiles in cannabis (Greenbaum 2019). The approach uses a two-compartment system in which one compartment contains an external source of volatiles and the other contains cannabis inflorescence. Henry’s Law describes how the external and native terpenes establish an equilibrium. The terpene content of the gas phase thus provides a replacement source for terpenes which have evaporated from the inflorescence.

To evaluate the effectiveness of the approach, inflorescence samples were stored in the presence of various external volatiles. Terpene “isolates”, terpene mixtures, and essential oils (EO) were utilized as external volatile sources. The terpene content of the inflorescence was quantitatively determined by headspace gas chromatography-mass spectrometry (HS GC-MS). Inflorescence preservation samples were analyzed for terpene content after different durations of storage and compared to initial and control. Repeatability and robustness of the approach were assessed by testing inflorescence from different cannabis chemotypes, DJ’s Gold (DjG) and Cream Caramel (Cre), as well as inflorescence from multiple harvests of the DjG chemotype. Due to age and processing conditions, the material from DjG chemotype was depleted of terpene content. The system’s ability to replenish the terpene content of depleted material was investigated. The ability of the approach to selectively adjust the terpene profile of the inflorescence was investigated by comparing the profile before and after storage in the presence of an 8-part terpene mixture. The developing method offers patients and recreational cannabis users the potential for inflorescence with a standardized terpene content, improving the reliability of the efficacy and experience.

## Methods

All experiments using cannabis were conducted at state licensed medical cannabis facilities in accordance with relevant state law and regulations. Cannabis samples from the DjG chemotype were obtained from Pennsylvania Medical Solutions (Vireo Health, PA). Experiments from Vireo Health, PA are referred to as Site One. Samples from two harvests were utilized: one harvest aged approximately 1 year (named “aged one-year”), and one harvest aged approximately 1 month (named “aged one-month”) post-harvest. The aged one-year DjG samples were ground by hand, the aged one-month DjG sample were finely ground by an industrial grinding machine. Nine samples were prepared, 4 from aged one-year and 5 from aged one-month DjG inflorescence. For the aged one-year group the samples were stored with the following external volatiles; 8-part terpene mixture, lavender EO, cinnamon bark EO, and control. The following samples were prepared for the aged one-month DjG group; 8-part terpene mixture, β-myrcene, α-pinene, argon gas, and control. 1 mL of the external volatile was added to a 1.5 mL glass vial and placed inside the jars containing the cannabis. The inflorescence samples were stirred, and 50 mg was obtained, for terpene quantitation by HS GC-MS at both 2 and 4 weeks. The profile of the 8-part terpene mixture is presented in the Supplemental Table I.

A second set of cannabis inflorescence samples of the Cre chemotype were obtained from MaryMed (Vireo Health, MD). Experiments from Vireo Health, MD are referred to as Site Two. Experiments were performed on intact Cre inflorescence. The initial terpene content of inflorescence from the Cre chemotype was assessed by quantitative terpene analysis of 0.5 g. Fifteen samples were prepared, each containing approximately 1.5 g of intact inflorescence (exact weight was recorded) from the Cre chemotype in individual 4-oz airtight jars. A sponge was used as an inert matrix and added to each airtight jar. The sponge was impregnated with terpenes of the following volumes: 0 μL (control), 10 μL, 100 μL, 500 μL, and 1000 μL. A terpene blend named “Granddaddy Purps” was used as the external volatile for Site Two experiments. When adjusting for density (0.84 g/mL) these volumes represent a terpene content of approximately 0, 0.56, 5.6, 28, and 56%, weight by weight as compared to the inflorescence. The inflorescence and the inert matrix were separated by a screen so no terpenes could be transferred to the inflorescence by contact. Three samples from each group were prepared resulting in 15 total samples. To mimic patient use, samples were opened twice/week. Each sample was designated a specific storage duration (2, 4, and 6 weeks) and the entire sample was ground and subjected to quantitative terpene analysis at the conclusion of the designated storage duration.

All terpenes utilized in this study were derived from botanical sources. The following terpenes were sourced from True Terpenes (Portland, OR); β-myrcene, β-caryophyllene, linalool, α-(−)-bisabolol, D-limonene, α-humulene, α-terpineol, β-pinene, and α-pinene. Lavender and cinnamon bark EOs were sourced from Nature’s Oil (Aurora, OH). A terpene blend named “Granddaddy Purps” was sourced from Eybna Terpenes, Givat Hen, Israel. The blend is considered a β-myrcene, β-caryophyllene codominant blend by standards described by the USP Cannabis Expert Panel (Sarma et al. 2020). 4-oz airtight jars were sourced from the Ball Corporation (Westminster, CO). The argon canister was sourced from Art (Chicago, IL). Cellulose sponges were obtained from 3 M (Saint Paul, MN).

Quantitative terpene analysis of cannabis samples from Site One were analyzed by Keystone State Testing Labs, Harrisburg, PA. A Shimadzu TQ-8050 NX HS GC-MS with LabSolutions version 4.45 software was used. Method parameters can be found in Supplemental Table II. A Restek (Bellefonte, PA) Rxi-624Sil MS column, catalog number 13868, was used. The scope of the quantitative method included 42 terpenes (see Supplemental Table III). The 42-part terpene standard was sourced from Spex Certiprep (Metuchen, NJ). 50 mg of cannabis was prepared in ≥99.8% methanol (VWR, Radnor, PA), and sealed in a headspace vial for analysis. Terpene analysis was performed in duplicate for each sample and the average was used for data analysis. Example calibration curves for individual terpenes are provided in Supplemental Figure I. Similarly, cannabis samples from Site Two were analyzed by Steep Hill, Columbia, MD by a GC-MS method quantitatively assessing 38 terpenes.

Analysis of variance (ANOVA), main effects analysis and other data processing was performed using Minitab (State College, Pennsylvania) version 19.2. Main effects analysis and ANOVA (reported *p*-values) were used to qualitatively and quantitatively (respectively) evaluate the impact of different variables on the novel’s system effectiveness.

## Results

### Site one

Inflorescence samples were obtained from two different harvests of DjG. Approximately 2 g of ground inflorescence were used per sample. For terpene preservation samples, a vial containing 1 mL of the external volatile was placed into the airtight jar containing the inflorescence. Control samples did not include an external volatile. A summary of all results, including the initial and final terpene content of the inflorescence after storage, characterized by HS GC-MS, is provided in Table 1. The initial terpene content of the aged one-year and aged one-month DjG ground inflorescence samples were determined to be 0.170 and 0.153%, respectively. A terpene content of 0.170% or 0.153% is typically considered low for inflorescence intended for distribution. These terpene levels can be explained by the fact that the DjG samples for this experiment were taken from ground inflorescence intended for extraction to obtain relevant cannabinoids. The processing and grinding methods used on this material may have reduced the terpene content.

**Table 1:** Summary of results

**Chemotype**	**DJ’s Gold**			
**Time Elapsed Post Harvest**	**Storage Duration**	**Sample Name**	**Inflorescence Terpene Content (%, w/w)**	**Percent Change of Terpene Content Compared to Initial**
**1 Year**	**Initial**	Initial	0.170	N/A
	**2 Weeks**	Control	0.191	+ 12.4%
		8-Part Terpene Mixture	0.278	+ 63.5%
		Lavender Essential Oil	0.193	+ 13.5%
		Cinnamon Bark Essential Oil	0.224	+ 31.8%
	**4 Weeks**	Control	0.154	−9.41%
		8-Part Terpene Mixture	0.398	+ 134%
		Lavender Essential Oil	0.469	+ 176%
		Cinnamon Bark Essential Oil	0.291	+ 71.2%
**1 Month**	**Initial**	Initial	0.153	N/A
	**2 Weeks**	Control	0.091	−40.5%
		8-Part Terpene Mixture	0.116	−24.2%
		β-Myrcene	0.115	−24.8%
		α-Pinene	0.314	+ 105%
		Argon Gas	0.093	−39.2%
	**4 Weeks**	Control	0.074	−51.6%
		8-Part Terpene Mixture	0.185	+ 20.9%
		β-Myrcene	0.249	+ 62.7%
		α-Pinene	0.910	+ 495%
		Argon Gas	0.076	−50.3%
**Chemotype**	**Cream Caramel**			
**Storage Duration**	**External Terpene Amount** **(% Relative to Inflorescence)**		**Inflorescence Terpene Content (%, w/w)**	**Percent Change of Terpene Content Compared to Initial**
**Initial**	N/A		1.49	N/A
**2 Weeks**	0%		1.33	−10.7%
	0.56%		1.43	−4.03%
	5.6%		2.24	+ 50.3%
	28%		2.93	+ 96.6%
	56%		6.12	+ 311%
**4 Weeks**	0%		0.74	−50.3%
	0.56%		0.85	−43.0%
	5.6%		1.48	−0.671%
	28%		2.18	+ 46.3%
	56%		3.82	+ 156%
**6 Weeks**	0%		1.86	+ 24.8%
	0.56%		2.11	+ 41.6%
	5.6%		2.19	+ 47.0%
	28%		3.21	+ 115%
	56%		3.98	+ 167%
**Average**	0%		1.31	−12.1%
	0.56%		1.46	−1.79%
	5.6%		1.97	+ 32.2%
	28%		2.77	+ 86.1%
	56%		4.64	+ 211%

After 4 weeks of storage the control samples had a terpene content of 0.154 and 0.074%, representing a loss of 9.41 and 51.6% terpene content, for the aged one-year and the aged one-month DjG, respectively. Conversely, the terpene content increased for all samples stored in the presence of an external volatile. The highest increase in terpene content (495%) was observed for the aged one-month DjG sample which was stored with α-pinene. When compared to controls, samples stored in the presence of an 8-part terpene mixture had a total terpene content 2.6 and 2.5 times larger, for aged one-year and aged one-month DjG, respectively.

The impact of terpene oxidation was investigated by displacing the air in the headspace of the container with argon. As illustrated in Table 1, argon replacement did not mitigate terpene loss of aged one-month DjG inflorescence. The sample labeled “argon gas” experienced approximately the same loss of terpene content as the control.

**Fig. 1:**
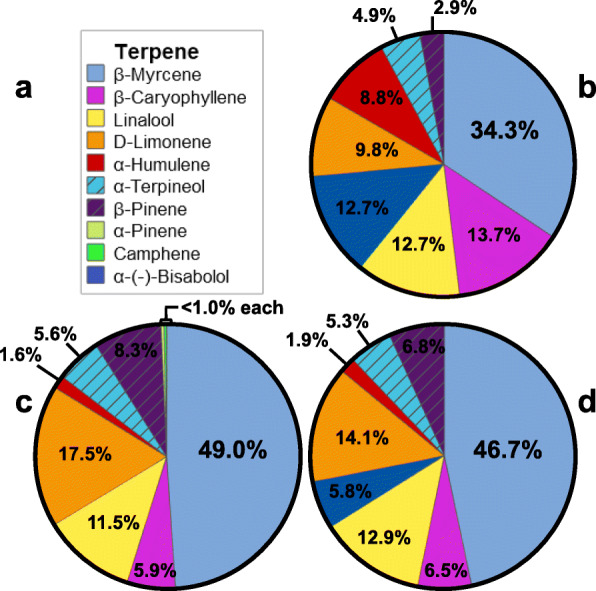
**a** Terpene key, **b** terpene profile of the external terpene mixture, **c** aged one-year DjG inflorescence, and **d** aged one-month DjG inflorescence after 4 weeks in the presence of the mixture

The accuracy and repeatability of modifying the inflorescence terpene profile using a formulated 8-part terpene mixture was investigated. The profile of the 8-part mixture is presented in Fig. 1b. The terpene profiles of the aged one-year DjG (Fig. 1c) and aged one-month DjG (Fig. 1d) inflorescence after 4 weeks of storage in the presence of the mixture are also presented. As illustrated in Fig. 1c the terpene profile of aged one-year DjG contained 7 of the 8 terpenes of the 8-part mixture, as well as two terpenes (α-pinene and camphene) which were present in the initial inflorescence. The terpene profile of the aged one-month DjG sample was successfully modified to contain only terpenes of the 8-part mixture (Fig. 1d).

The accuracy of establishing a specific terpene profile, of specific terpene ratios, in inflorescence samples was assessed. Inflorescence samples were stored in the presence of a formulated 8-part terpene mixture for 4 weeks. After 4 weeks of storage, each terpene’s percentage of the total terpene profile was calculated in the inflorescence samples and compared to the 8-part mixture. For example, β-myrcene represented 34.3% of the total terpene content of the 8-part mixture. After 4 weeks of storage in the presence of the mixture, the aged one-year and aged one-month DjG inflorescence had a β-myrcene content consisting of 49.0 and 46.7% of the total terpene content. The average terpene profile recovery for β-myrcene was calculated to be 139%. This recovery is graphically illustrated in Fig. 1, in which the light blue pie slices (representing β-myrcene’s percentage of total terpene content) are larger for the inflorescence samples as compared to the 8-part terpene mixture. The individual terpene recoveries are reported in Table 2. The terpenes used in the 8-part mixture can be separated into three categories: monoterpenes, monoterpene alcohols, and sesquiterpenes. Due to the sample size, the sesquiterpene alcohol, α-(−)-bisabolol, is grouped with the sesquiterpenes. As illustrated in Table 2, the percent recovery of the individual terpenes appears dependent upon the terpene class, monoterpenes having the highest rate of infusion, followed by monoterpene alcohols, and sesquiterpenes having the lowest rate.

**Table 2:** Terpene profile recoveries for each terpene in the DjG preservation samples. Recoveries calculated by comparing results to theoretical value of the formulated 8-part mixture to inflorescence terpene profiles after storage in the presence of the mixture

Terpene	Average % recovery	Terpene classification
β-Pinene	257	Monoterpene
D-Limonene	161	
β-Myrcene	139	
α-Terpineol	111	Monoterpene alcohol
Linalool	95.7	
β-Caryophyllene	45.2	Sesquiterpene
α-(−)-Bisabolol	22.8	
α-Humulene	19.8	

The repeatability of the approach was assessed by comparing the terpene profile of the aged one-year and aged one-month DjG samples before and after storage in the presence of the 8-part mixture. Inflorescence from the two harvests of DjG had an initial percent difference in terpene profile of 55.8%, calculated by the weighted mean of the percent difference for all terpenes (see Supplemental Table IV). After 4 weeks of storage in the presence of the 8-part mixture, the percent difference was reduced to 16.3% (see Supplemental Table V), an improvement of 39.5%. The remaining 16.3% variation mainly originated from the fact that α-(−)-bisabolol (Fig. 1 dark blue slices) was not infused into the aged one-year DjG. Conversely, α-(−)-bisabolol represented 5.8% of the aged one-month DjG terpene profile after storage in the presence of the mixture. When α-(−)-bisabolol is considered an outlier, the variance between the two inflorescence samples is reduced to 10.8%.

**Fig. 2:**
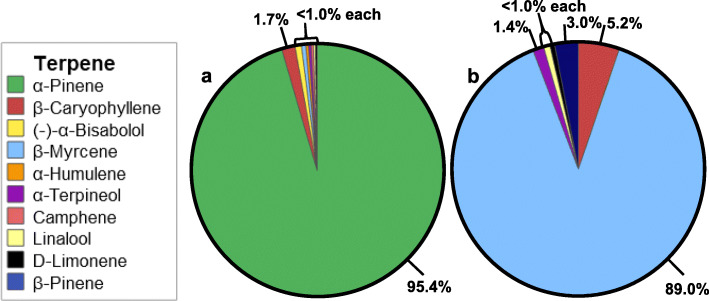
Terpene profile of aged one-month DjG samples stored in the presence of α-pinene (**a**) and β-myrcene (**b**) “isolates”

Inflorescence terpene profiles were also selectively adjusted to be dominated by individual terpenes. Inflorescence with terpene profiles consisting of 95.4% α-pinene (Fig. 2a), as well as 89.0% β-myrcene (Fig. 2b), were achieved when inflorescence was stored with the corresponding isolated terpene. Terpenes not native to the DjG chemotype were also successfully infused into DjG inflorescence. Lavender and cinnamon bark EOs were used as external volatile sources. The 2 most abundant terpenes in the lavender EO (linalool and ocimene), were also the two most abundant terpenes in the cannabis inflorescence after storage with the EO. The cinnamon bark EO was able to introduce 5 terpenes that were not present in the initial terpene profile for the aged one-year DjG inflorescence. The cannabis inflorescence obtained the aroma of the EOs after storage.

### Site two

**Fig. 3:**
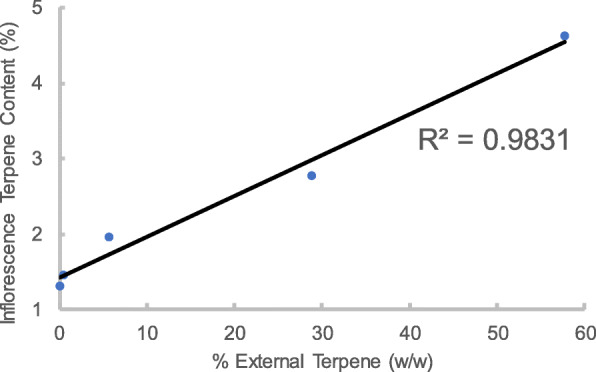
Terpene content of preservation samples versus amount of external terpene. Linear best fit line and coefficient of determination (R^2^) are displayed

Additional validation studies were performed on inflorescence from the chemotype Cre. The initial terpene content of the Cre inflorescence was determined to be 1.49%. For preservation samples, a terpene blend named “Granddaddy Purps” was impregnated into an inert matrix and placed into an airtight jar containing the inflorescence. Varying amounts of terpenes were impregnated into the sponge, ranging from 0.5 to 57.8% of the weight of the inflorescence sample. The inert matrix for the control samples did not contain terpenes. Samples were prepared in triplicate for the 5 terpene concentrations, representing 15 preservation samples. Each sample was opened twice per week to mimic patient use. Samples were stored for 2, 4, and 6 weeks for each terpene concentration. The average loss of terpene content for the controls was 12.1%, calculated by averaging the three samples. For preservation samples using 1 mL of external volatile, a 3.5-times increase in terpene content as compared to control was observed. Figure 3 illustrates the trend in inflorescence terpene content versus amount of external terpene utilized, each datapoint is an average of the three samples. Using the equation of the line, the percent of external terpene (relative to amount of inflorescence) required to maintain the native terpene content of the initial inflorescence was determined to be 1.18%. The coefficient of determination (R^2^) for the linear fit was 0.9831.

The change in terpene profile over time a 2-week period was assessed. Terpene profiles from samples of Cre stored in the absence and presence of an external volatile were compared. Control samples after 2 and 4 weeks of storage were found to have a percent difference in terpene profiles of 41.9% (Supplemental Table VI). Conversely, when comparing the terpene profiles of two separate samples after 2 and 4 weeks of storage in the presence of an external volatile, the percent difference was 19.5% (Supplemental Table VI). Explicitly, the use of the novel system maintains terpene profile during storage.

Inflorescence samples from both Site One and Site Two experienced a similar increase in terpene content when 1 mL of external volatile was utilized (Table 3). The increase in terpene content (as compared to control) versus the percent external volatile utilized were calculated. The resulting ratio was approximately 6 for all three inflorescence groups. The range of ratios obtained were 5.9–6.1, representing a 3.4% variance across the range.

**Table 3:** Summary of the terpene content increase for preservation samples utilizing 1 mL (840 mg) of external volatile

Sample	Amount inflorescence (mg)	Amount external terpene (mg)	% External terpene relative to inflorescence	Increase in terpene content versus control	Ratio terpene content increase:%external terpene
**Aged One -Year DjG**	1950	840	43.1%	2.6x	6.0
**Aged One-Month DjG**	1980	840	42.4%	2.5x	5.9
**Cre**	1460	840	57.8%	3.5x	6.1

**Fig. 4:**
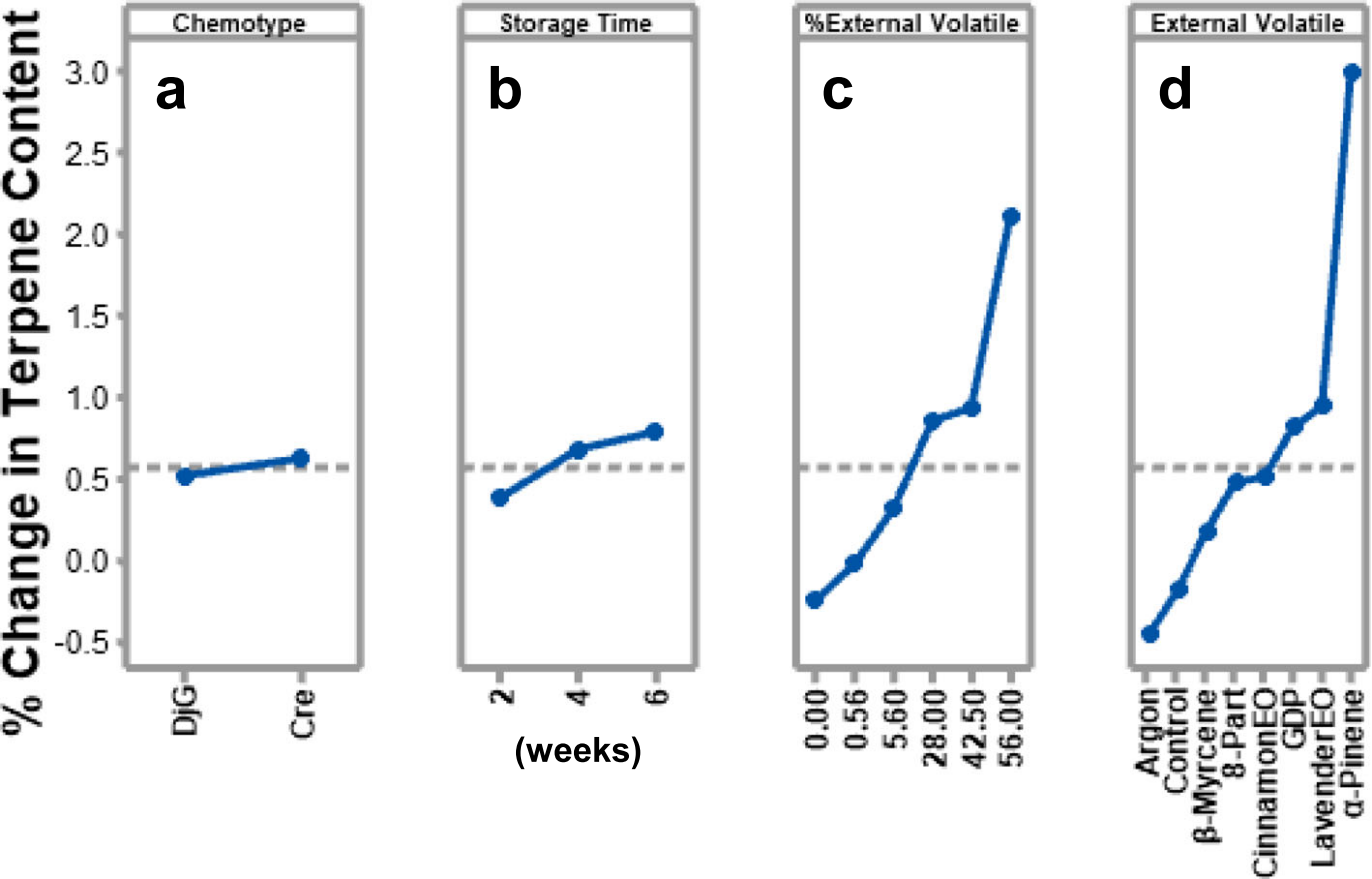
Main effects plot evaluating the impact of each variable on the system’s effectiveness towards terpene preservation. **a** Chemotype of inflorescence **b** storage time of inflorescence **c** percent external volatile relative to inflorescence **d** different external volatiles utilized, GDP = Granddaddy Purps terpene blend

The robustness of the novel system was evaluated by testing the effectiveness of the system for preserving terpenes in cannabis inflorescence under various conditions. Four variables were analyzed to determine if they had a statistically significant impact on the novel system; chemotype, storage duration, percentage external volatile utilized, and external volatile type. Data generated from both sites was subjected to main effects analysis (Fig. 4). The y-axis of the plot in Fig. 4 illustrates the mean percent change in terpene content over the storage duration compared to initial, while the x-axis plots the various parameters. Site One percentage of external volatiles utilized (42.5%) was obtained by average all Site One samples. The slope of the plots qualitatively corresponds to statistical significance. For example, a plot with no change in slope indicates that the result does not change with different variables, thus the variable is less likely to have a statistically significant influence on the system (Fig. 4a and b). Conversely, percent external volatile and external volatile type show high correlation between altered variables and system effect. ANOVA was performed to determine if the mean percent change between groups was statistically significant. Variables with reported *p*-values of < 0.05 were considered significant, as this indicated the mean values of each factor were not equivalent. Results are reported in Table 4 and illustrate that chemotype and storage time have *p*-values of > 0.05 while percent external volatile and external volatile type have *p*-values < 0.05. Thus, chemotype and storage duration are not considered statistically significant while percent external volatile and external volatile type are considered statistically significant towards the effectiveness of the system.

**Table 4:** Resulting *p*-values from ANOVA models evaluating the impact of each variable on the system’s effectiveness. A *p*-value of < 0.05 is considered statistically significant

Variable	*p*-value
Chemotype	0.793
Storage Time	0.715
%External Volatile	0.013
External Volatile	0.015

## Discussion

Patients often source cannabis inflorescence for medical purposes in one-month doses. Additional flexibility exists for consumers in adult use markets, but storage of inflorescence over one-month periods is not uncommon. Since the entire inflorescence sample is not consumed in one dose, patients must open and close the storage container numerous times to obtain a portion of the inflorescence. Continually opening the container allows terpenes which have evaporated from the inflorescence to escape the storage container and contributes to reduction of inflorescence terpene content over time. Other sources which can affect the terpene content of inflorescence include cultivation, harvest, storage, and curing (drying) methods, chemotype genetics, as well as storage conditions and age of the inflorescence. These factors present challenges for patients and consumers seeking inflorescence of reliable terpene content.

A novel method to preserve, replenish, and establish a desired terpene content of cannabis inflorescence was investigated. A total of 38 inflorescence samples were investigated in this study (Table 1). Nine samples were stored without an external volatile source (controls) while 27 samples were stored in the presence of an external volatile. Eight of the nine controls samples experienced a loss in terpene content over the duration of storage. The aged one-year DjG control unexpectedly experienced an increase in terpene content after 2 weeks of storage as compared to initial, which may have been caused by sample heterogeneity. It should be noted that the control samples from the two harvests of DjG experienced different rates of terpene loss. The aged one-year DjG sample experienced a 9.41% loss while the aged one-month DjG sample experienced a 51.6% loss of terpene content after 4 weeks of storage. It is likely the aged one-month DjG had a higher percentage of more volatile terpenes as compared to the aged one-year DjG. Thus, the aged one-month DjG was more susceptible to terpene loss in the experiments described here. The differing rates of terpene loss from inflorescence of the same chemotype, but different harvests, is further illustrative of the inherent variability of cannabis inflorescence terpene content.

The repeatability and robustness of the approach was investigated by testing the system using different chemotypes, storage times, percentages of external volatiles, and types of external volatiles. The ‘Chemotype’ group also included differences in inflorescence cultivation locations, different harvests and age, ground versus intact inflorescence, initial terpene content (typical versus depleted), and neat volatiles stored in a glass container versus volatiles impregnated into an inert matrix. Furthermore, quantitative terpene analysis for the different sites was performed by different testing labs, utilizing different analysts. Experiments at Site Two mimicked patient use by opening the storage jars twice per week. Under all conditions, all preservation samples (*n* = 27) successfully maintained a higher terpene content as compared to controls after 2 and 4 weeks (Site One) and 2, 4, and 6 weeks (Site Two) of storage, illustrating the robustness of the method. Variables that were statistically meaningful to the effectiveness of the novel system were identified by main effects analysis and ANOVA modeling. The amount and type of external terpene utilized were found to be statistically meaningful parameters towards the effectiveness of the system for terpene preservation and augmentation. Conversely, chemotype and storage time did not impact the novel’s system ability to preserve terpenes. Explicitly, all factors that were varied in the chemotype group, most notably the initial terpene content of the inflorescence, did not impact the method’s ability to preserve or augment the terpene content of the inflorescence, further illustrating the robustness of the novel system. All durations of storage were found to be effective towards terpene preservation, illustrating that the maximum storage duration has not been identified, however as results indicate it is greater than 6 weeks.

As described previously, samples from the DjG chemotype were almost entirely depleted of terpene content (0.153 and 0.170%) due to the processing and grinding methods applied to the inflorescence post-harvest. Conversely, samples from the Cre chemotype had an initial terpene content of 1.49%. This terpene content is within the expected range for inflorescence available for medicinal or recreational use (Jin et al. 2020). For both sample groups the novel system was able to increase the terpene content of the inflorescence, illustrating the system is effective independent of initial terpene content of the material. Results illustrate the novel system was able to replenish the terpene content of the DjG inflorescence samples. The largest augmentation was achieved by using α-pinene resulting in an increase from 0.153 to 0.910% terpene content for the inflorescence samples. Although 0.910% inflorescence terpene content would be regarded as lower than what is typical for medicinal or recreational use, the + 495% change illustrates a proof of concept for the system’s ability to replenish terpenes from terpene depleted inflorescence. Figure 3 illustrates that the degree of inflorescence terpene content augmentation is directly related to the amount of external terpene is used. In this study terpene content augmentation was maximized by storing inflorescence samples in the presence of external volatiles representing ~ 42–58% the weight of the inflorescence. However, larger percentages could be utilized to further augment the terpene content of both terpene containing or terpene depleted inflorescence. Thus, providing patients and manufacturers the ability to establish a desired terpene content for their inflorescence. The rate of terpene augmentation, relative to the percent external volatile utilized, was similar for all inflorescence groups (Table 3). When adjusting for the weight of the individual inflorescence samples, the terpene content augmentation was within 3.4% for all three inflorescence groups, illustrating the reproducibility of the method.

The repeatability of the approach to selectively modify the inflorescence terpene profile was investigated. Inflorescence samples from different harvests were stored in the presence of a formulated 8-part terpene mixture. The percent difference of the terpene profiles was compared before and after storage. Preliminary results indicate a 39.5% reduction in terpene profile variance for the different inflorescence samples of the DjG chemotype. It should be noted that the approach was effective even in the extreme scenario investigated in this study, in which the inflorescence samples differed in age by approximately 1 year. Similar terpene profiles obtained in cannabis inflorescence samples from two independent harvests, separated by 1 year, illustrates a proof of concept for improving batch to batch consistency of terpene content cannabis inflorescence. Site Two experiments showed that variability in the terpene profile caused by aging, and different rates of terpene loss, of inflorescence is reduced when using the novel system. This development may address a common real-world scenario, in which patients and consumers source inflorescence of the same chemotype at different periods of time. Patients and consumers may expect reliable terpene profiles (which is often correlated to efficacy and experience) from inflorescence of the same chemotype. However, as illustrated in this work and previous research, the terpene profiles of the inflorescence may vary due to age, batch (harvest), and other factors. Results reported here illustrate the variance between inflorescence samples can be significantly reduced when the novel packaging approach is utilized.

In this proof of concept study, botanically derived terpenes were utilized as the external volatile source. However, methods for extracting terpenes from cannabis are well established and terpenes extracted from cannabis could be reintroduced to the inflorescence using the novel system described here. Similar to preliminary results, utilizing the novel system with chemotype specific terpenes as the ‘external’ volatiles, is expected reduce terpene loss over time, and batch to batch variability of the terpene profile. It is worth noting that any terpenes or other volatiles utilized as an external volatile source, requires control of potential manufacturing impurities which could be introduced into the inflorescence. The reintroduction of chemotype specific terpene profiles may provide a path for pharmaceutical development via the FDA’s Botanical Drug pathway, as all ingredients would originate from the same botanical (cannabis) source, with the improved batch to batch consistency offered by the novel system.

The accuracy of adjusting the terpene profile to match a formulated blend was assessed by comparing the recoveries of each terpene in the inflorescence after storage, to the terpene profile of the formulated blend. The recoveries were found to be dependent upon terpene class, monoterpenes which contain 2 isoprene units (C_10_H_15_) were observed to have the highest rate of terpene infusion as compared to sesquiterpenes, which contain 3 isoprene units (C_15_H_24_). Both isomers of the monoterpene pinene had the highest rate of terpene infusion for the 8 terpenes investigated (Table 2) and produced inflorescence with a higher terpene content as compared to other external volatile sources (Table 1 and Fig. 2a). These factors may indicate that terpene volatility, in which monoterpenes are more volatile as compared to sesquiterpenes, determines the rate of infusion. Characterizing the infusion rates of various terpenes is a future direction for this research. Conversely, the ability to selectively adjust the terpene profiles of inflorescence samples to be dominated by individual terpenes was achieved with an accuracy of 95.4% for α-pinene and 89.0% for β-myrcene.

Although further clinical research would be required to satisfy the medical and scientific community, there is widespread popular belief that, different chemotypes of cannabis are associated with specific pharmacological effects. The characteristic terpene profiles and total terpene content of various cannabis chemotypes may be responsible for the differences in perceived pharmacological and medicinal benefits (Hazekamp et al. 2016; Mudge et al. 2019; Lewis et al. 2018). For example, inflorescence with a terpene profile dominated by β-myrcene is often associated with calming or sedative effects (Sarma et al. 2020). Similarly, an α-pinene dominant terpene profile may have efficacy towards treating anxiety, as the inhalation of α-pinene has been linked to anxiolytic-like activity in mice and rats (Satou et al. 2014; Zhang and Yao 2019). Due to cost and availability, the pulmonary delivery of cannabis inflorescence (either through vaporization or combustion) is the main delivery method for both medicinal and recreational cannabis users. The so-called ‘entourage effect’, or the concept that whole plant products have improved efficacy as compared to their individual chemical constituents, is the proposed benefit of this delivery form (Russo 2011). Further research on the entourage effect is required, but recent research has been shown that pulmonary delivery of whole plant cannabis products may be more advantageous for treating specific pain types (neuropathic), as compared to other routes of cannabinoid absorption, such as oromucosal (Rabgay et al. 2020). However, variability and/or loss of inflorescence terpene content may reduce the enhanced cannabinoid pharmacology associated with the entourage effect. Variability in cannabinoid-terpene synergism may lead to unreliable experience and efficacy of medicinal and recreational cannabis inflorescence. Achieving batch to batch consistency and stability of all phytochemical components, including terpenes, is an important step towards the pharmaceuticalization of cannabis inflorescence and an unmet need in the cannabis industry (Koltai et al. 2019). The novel system described here addresses the drawbacks associated with terpene loss from inflorescence over time and has the potential to improve experiences for both medical and recreational cannabis users.

Experiments reported in this manuscript assessed the practicality and cost of the novel packaging system. It was experimentally determined that approximately 41.3 mg of external terpenes are required to maintain the native terpene content of 3.5 g of inflorescence (a common amount available at dispensaries). Since terpene extraction methods from both cannabis and botanical sources are well established, and can be achieved at relatively large scales, 41.3 mg of terpenes adds minimal cost to a final product. Several inert matrices of sizes ≤1 cm^3^ are available which are capable of housing this volume of terpenes. Finally, the compartment which houses the terpene impregnated matrix can be incorporated directly into current inflorescence packaging. Thus, the authors expect the components of the novel system to add minimal cost to individual inflorescence sale units. The practicality of the novel system is improved by the fact that manufacturers and consumers are not required to handle neat terpenes, as positive results were obtained from inflorescence samples stored in the presence of terpenes impregnated into an inert matrix.

## Conclusions

The inherent volatility of terpenes often results in loss of terpene content from cannabis inflorescence over time. Unlike cannabinoids, which do not share the same volatility, and can be preserved through cultivation and processing practices, there is no readily available method for the preservation of terpenes in cannabis inflorescence. Reported here is the successful proof of concept towards the standardized control of terpene content in cannabis inflorescence, the preservation of native terpene content, selective augmentation and adjustment of inflorescence terpene profiles, and the ability to adjust the aroma of the inflorescence. Several validation parameters for the novel packaging approach were achieved in this study. Robustness was achieved by obtaining positive results for both ground and intact inflorescence, and when utilizing both neat external volatiles and external volatiles impregnated into a matrix. Intermediate precision was achieved by obtaining positive results from inflorescence at different sites, chemotypes, and analytical characterization labs. Similar results were achieved for terpene augmentations, even when utilizing different external volatiles and inflorescence from different chemotypes.

Preliminary results indicate that the fundamental applications of the novel system include maintaining a desired native terpene content of the inflorescence at the time of packaging and the replenishment of terpenes to terpene-depleted inflorescence. More sophisticated applications that require further investigation include adjusting the flavors, aromas, and potentially the pharmacological effects of inflorescence by selective adjustment of the inflorescence terpene profile. The application of adjusting the aroma of cannabis inflorescence, as illustrated in this study when stored in the presence of lavender EO, may reduce the stigma of inflorescence use and increase the number of patients willing to access the medicinal benefits of whole plant products. For example, patients may be more apt to use cannabis inflorescence which possesses a lavender aroma as compared to the typical inflorescence aromas, which often have a negative association with non-cannabis users.

Future directions of this research include determining the maximum storage time terpenes can be preserved, further investigating the efficiency of absorption of various terpenes, and utilizing the natural antimicrobial properties of terpenes towards inhibiting the growth of bacterial and/or fungal contaminants on inflorescence.

## Supplementary information

**Additional file 1.**

## Data Availability

Data presented in this work is available via a reasonable request to the corresponding author.

## Abbreviations

ANOVA: Analysis of variance
Cre: Cream Caramel
DjG: DJ’s Gold
EO: Essential oil
GDP: Granddaddy Purps
HS GC-MS: Headspace gas chromatography-mass spectrometry
N/A: Not applicable
R^2^: Coefficient of determination
w/w: Weight/Weight
