# Supplementary material for: The preservation and augmentation of volatile terpenes in cannabis inflorescence
Source: J Cannabis Res. 2020 Sep 14;2:27. doi: 10.1186/s42238-020-00035-z (PMC7819294; doi:10.1186/s42238-020-00035-z)
Supplement: Supplementary file 1 — Additional file 1. [file 42238_2020_35_MOESM1_ESM.pdf]

The preservation of volatile terpenes in cannabis inflorescence

Justin Bueno, Emily Leuer, Michael Kearney Jr., Edward H. Green, Eric A. Greenbaum

Supplemental Materials

Supplemental Table I. Profile of external terpene mixture

Terpene	%(w/w)
β-Myrcene	34.3
β-Caryophyllene	13.7
Linalool	12.7
α-(-)-Bisabolol	12.7
D-Limonene	9.8
α-Humulene	8.8
α-Terpineol	4.9
β-Pinene	2.9

Supplemental Table II. Site One instrument parameters

HS Parameter	Setting
Oven Temperature	150˚C
Sample Line Temperature	150˚C
Transfer Line Temperature	150˚C
Pressurizing Gas Pressure	50.0 kPA
Equilibrating Time	30 min.
Pressurizing Time	1 min.
Pressure Equilibrium Time	0.2 min.
Load Time	1 min.
Load Equilibration Time	0.2 min.
Injection Time	1.0 min.
Needle Flush	1.0 min.
**GC Parameters**	**Setting**
Oven Temperature	80˚C
Injection Mode	Split
Carrier Gas	He
Flow Control Mode	Pressure
Pressure	92.9 kPA
Total Flow	17.4 mL/min
Column Flow	1.31 mL/min
Linear Velocity	42.2 cm/sec
Purge Flow	3.0 mL/min

Supplemental Table III. Site One terpene scope of the HS GC-MS method. 42-part standard contained all terpenes at 100 µg/mL

**Terpene**	**CAS#**
α-(-)-Bisabolol	23089-26-1
(-)-Caryophyllene Oxide	1139-30-6
(-)-Isopulegol	89-79-2
(+)-Cedrol	77-53-2
(+)-Fenchone	4695-62-9
(1S)-(+)-3-Carene	498-15-7
β-Myrcene	123-35-3
Camphene	79-92-5
Camphor	76-22-2
cis-Nerolidol	3790-78-1
Eucalyptol	470-82-6
Farnesene (mix of isomers)	502-61-4
Geranyl Acetate	105-87-3
Hexahydrothymol	89-78-1
Isoborneol	124-76-5
Linalool	78-70-6
Nerol	106-25-2
Ocimene (Mixture of Isomers)	13877-91-3
p-Mentha-1,5-diene	99-83-2
β-Caryophyllene (trans-Caryophyllene)	87-44-5
Valencene	4630-07-3
(-)-Borneol	464-45-9
(+)-Borneol	464-43-7
(+)-Pulegone	89-82-7
(1R)-(+)-Camphor	464-49-3
(1R)-Endo-(+)-Fenchyl Alcohol	2217-02-9
(1S)-(-)-Camphor	464-48-2
(R)-(+)-Limonene (D-Limonene)	5989-27-5
α-Cedrene	469-61-4
α-Humulene	6753-98-6
α-Terpinene	99-86-5
α-Pinene	80-56-8
β-Pinene	127-91-3
γ-Terpinene	99-85-4
Geraniol	106-24-1
Guaiol	489-86-1
L(-)-Fenchone	7787-20-4
Sabinene	3387-41-5
Sabinene Hydrate	546-79-2
Terpineol (mixture of Isomers)	8000-41-7
Terpinolene	586-62-9
trans-Nerolidol	40716-66-3

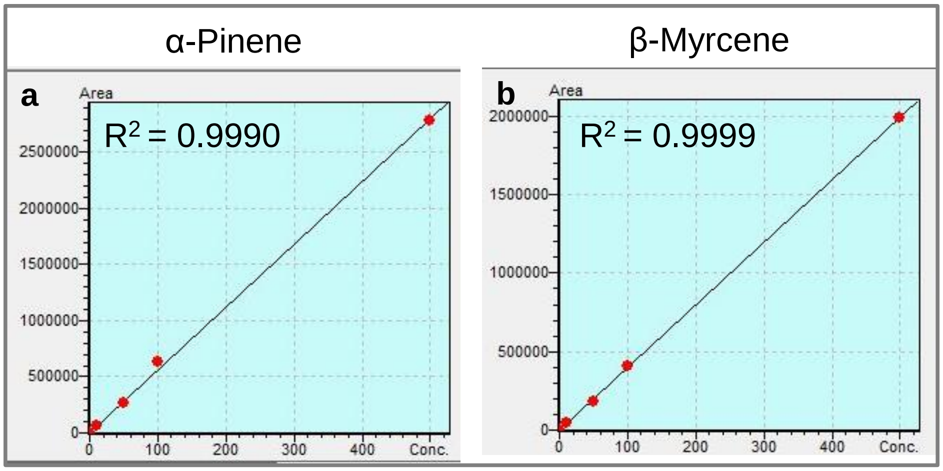

Supplemental Figure 1. Calibration curves used for quantitative determination of α-pinene and β-myrcene from Site One experiments

Supplemental Table IV. Initial terpene profile of aged one-year and aged one-month DjG samples and their differences in terpene composition

Terpene	% of Profile		% Difference
	Aged One-Year DjG	Aged One-Month DjG	
β-Myrcene	13.8	36.0	89.4
β-Caryophyllene	21.5	23.8	10.3
D-Limonene	14.3	15	4.6
Valencene	4.1	6.8	48.7
β-Pinene	8.0	5.6	35.1
α-Humulene	6.0	4.7	23.7
Linalool	11.7	3.8	101.8
α-Pinene	9.1	3.3	93.2
Camphene	2.0	1.0	69.7
α-Terpineol	6.2	0.0	200
β-Farnesene	1.9	0.0	200
Trans-Nerolidol	1.6	0.0	200
Average			89.7
Weighted Average			55.8

Supplemental Table V. Terpene profile after four weeks of storage in the presence of the 8-part mixture for aged one-year DjG and aged one-month DjG samples and their corresponding % difference

Terpene	% of Profile		% Difference
	Aged One-Year DjG	Aged One-Month DjG	
β-Myrcene	49	46.7	4.8
β-Caryophyllene	5.9	6.5	9.7
Linalool	11.5	12.9	11.5
α-(-)-Bisabolol	0.0	5.8	200
D-Limonene	17.5	14.1	21.5
α-Humulene	1.6	1.9	17.1
α-Terpineol	5.6	5.3	5.5
β-Pinene	8.3	6.8	19.9
α-Pinene	0.4	0.0	200
Camphene	0.3	0.0	200
Average			69.0
Weighted Average			16.3

Supplemental Table VI. Comparing agreement of terpene profiles from Cream Carmel (Cre) samples. Terpene profile compared at two and four weeks of aging for samples stored in the absence and presence of external volatiles. Samples stored in the presence of external volatiles are an average of the four external terpene concentrations. Corresponding % difference between the samples are reported

Terpene	Cre Sample, % of Profile		% Difference	Cre Sample, % of Profile		% Difference
	2 Week - Control	4 Week - Control		2 Weeks – With External Terpenes	4 Weeks – With External Terpenes	
α-Pinene	3.0	4.1	29.6	12.9	14.4	10.6
Camphene	1.5	1.4	10.7	1.0	1.5	34.7
β-Pinene	3.0	4.1	29.6	4.4	4.7	7.6
β-Myrcene	4.5	5.4	18.0	12.3	8.1	41.6
(1S)-3-Carene	0.0	0.0	N/A	0.1	0.0	200
D-Limonene	18.0	28.4	44.5	17.2	18.7	8.3
Ocimene	4.5	0.0	200	3.2	0.0	200
Sabinene Hydrate	0.0	0.0	N/A	0.6	0.6	0.4
Terpinolene	0.0	0.0	N/A	0.3	0.3	15.5
Linolool	6.0	5.4	10.7	12.8	13.5	5.7
Fenchol	3.8	5.4	35.9	2.4	2.4	0.2
Terpineol	5.3	6.8	24.9	3.2	3.5	8.1
Nerol	1.5	0.0	200	0.6	0.0	200.0
Geraniol	2.3	1.4	50.1	1.2	0.6	56.7
β-Caryophyllene	24.1	18.9	23.9	13.2	12.7	4.0
α-Humulene	7.5	5.4	32.7	2.1	1.9	13.6
Valencene	1.5	0.0	200	0.3	0.1	79.9
trans-Nerolidol	1.5	0.0	200	0.0	0.0	N/A
Caryophyllene oxide	1.5	0.0	200	6.0	10.5	54.1
Guaiol	0.0	2.7	200	1.5	1.3	11.3
β-eudesmol	2.3	4.1	57.0	1.8	1.9	9.6
α-(-)-Bisabolol	8.3	6.8	20.1	3.0	3.3	10.1
Average			83.6%	Average		46.3%
Weighted Average			41.9%	Weighted Average		19.5%

N/A = Not applicable. Terpenes not present in the profile were not averaged.
